# EARLY TEAM BASED NEURO-REHABILITATION AFTER MILD TRAUMATIC BRAIN INJURY: A PILOT STUDY

**DOI:** 10.2340/jrm-cc.v8.44355

**Published:** 2025-10-19

**Authors:** Ann-Charlotte Lindström, Katharina Stibrant Sunnerhagen, Lena Nordeman

**Affiliations:** 1 Research, Education, Development and Innovation primary care, Region Västra Götaland, Sweden; 2 Närhälsan Rehabilitation Sörhaga, Alingsås, Sweden; 3 Section for Clinical Neuroscience, Institute of Neuroscience and Physiology, University of Gothenburg, Gothenburg, Sweden; 4 Hospital Rehabilitation medicine, Sahlgrenska University hospital, Gothenburg, Sweden; 5 Rehabilitation and Health, Institute of Neuroscience and Physiology, University of Gothenburg, Sweden

**Keywords:** anxiety, activities of daily living, fatigue, patient reported outcome measures, pilot project, post-concussion syndrome, neurological rehabilitation

## Abstract

**Objective:**

Evaluate study design, procedure, and measurements for future study of early rehabilitation after mild traumatic brain injury.

**Design:**

A randomized controlled study was conducted.

**Subjects/patients:**

Patients from a county hospital emergency department, diagnosed with mild Traumatic Brain Injury were contacted 2 weeks post-trauma.

**Methods:**

Patients who met the inclusion criteria were randomized into 2 groups (*n*=28). The intervention group received early rehabilitation from a team consisting of physio- and occupational therapists. The control group received usual care. Patient-reported outcomes for measures were fatigue, anxiety and depression, health-related quality of life, physical and activity levels, and sleep after trauma. Data were collected 3 and 16 weeks after trauma.

**Results:**

Patient-reported outcomes measures showed improvement in both groups for symptoms, physical and activity levels, sleep quality and quantity. Also, improvement in the sub-scales of fatigue and health- related quality of life but worsening for general fatigue and general health at post-test in both groups. Neither group showed improvement for anxiety or depression: the intervention group rated depression higher, and the control group rated anxiety higher, post-test.

**Conclusion:**

Design, procedures, measurements and interventions were feasible but need refinement for a full-scale study.

## LAY ABSTRACT

This pilot study explored whether the design and methods for a future full scale randomized controlled trial on early rehabilitation after mild traumatic brain injury would be feasible. Patients diagnosed with mild traumatic brain injury at a county hospital were contacted 2 weeks post injury. Twenty-eight participants were randomly assigned to either early rehabilitation with physiotherapists and occupational therapists or to treatment as usual. They completed questionnaires on fatigue, anxiety and depression, health-related quality of life, physical and activity levels, and sleep habits at 3- and 16 weeks post injury. Both groups showed improvement in symptoms, activity levels, and sleep. However, general fatigue and overall health worsened, and there was no improvement in symptoms of anxiety or depression. Depression increased in the early rehabilitation group, while anxiety increased in the control group. The study methods were feasible but require some adjustments before a full-scale trial.

In Sweden, approximately 10,000 people receive hospital care, and 20,000 primary care, for traumatic head injury (TBI) (1). Approximately 80% of the TBIs are reported as mild and there are a gender differences in the epidemiology were male are overrepresented with 73% (2). To be classified as mild traumatic brain injury (mTBI) by the Glasgow Coma Scale the criteria are: score 13–15, loss of consciousness 0–30 min, alteration of consciousness for up to 24 h, and normal imaging (1–3). A concussion is often considered a subset of mTBI and these terms are frequently used interchangeably (4).

In Sweden, 2602 patients with mTBI from 39 hospitals participated in a 3-month follow-up using the Rivermead Post-Concussion Symptoms Questionnaire (RPQ) (5). Most had no persistent symptoms, but 25% reported 3 or more, and 10% reported seven or more symptoms (6). Common symptoms after mTBI are headache, dizziness, blurred vision, fatigue, memory and concentration difficulties, irritability, restlessness, depression and reduced stress tolerance (7). The majority of patients with mTBI will recover within 1 to 2 weeks (2).

Prognostic factors for symptoms persisting a year or more have been suggested as; more severe symptoms in the acute phase, previous psychiatric history, previous mTBI, injury caused by assault, pre-injury unemployment, or inability to return to work 6 weeks post-injury (8). Symptoms lasting longer than 3 months can be classified as post-concussion syndrome (PCS) (9). Most of the PCS could be viewed as common reactions to the stress of injury or other mental and physical health stressors (9, 10). Persons with mTBI reported a greater degree of PCS symptoms 3 months post-injury, and there were no differences found 1 year after injury at follow-up (11). A prospective study (12) found that, 1 year post-injury, the most common new psychiatric disorders were depression and generalized anxiety (12). In another Swedish long term (7–8 years) follow-up study (13) a third of 595 patients reported residual post-TBI symptoms. A higher percentage of women reported symptoms. One-third of patients with repeated head trauma did not fully recover compared to one-fifth of those with a first TBI (13).

Rehabilitation should be customized according to severity. For mild brain injury there is strong evidence for the benefit of verbal and written information following hospital care (14). Traditionally, consensus-based recommendations have emphasized strict physical and cognitive rest after mTBI until symptom resolution (15, 16) and that gradual return to physical activity after trauma should be at a symptomatic level (17). Later studies have demonstrated that physical activity is beneficial in decreasing PCS in both the acute and chronic phases after mTBI. There are also results indicating that aerobic activity at a unimodal sub-threshold may be the best course of action compared with multimodal interventions (18).

Specialized brain injury rehabilitation, with a focus on problem-solving therapy or cognitive behavioural therapy (CBT), has shown effects in reducing residual symptoms, improving psychological functioning, alleviating depressive symptoms, and enhancing activity levels, participation, and health-related quality of life, when compared to care as usual (1). The report states 2 recommendations for rehabilitation after mTBI; problem-solving therapy or CBT, even if the evidence is low, and specialized interdisciplinary rehabilitation, which they recommend more research about.

A randomized controlled study (RCT) (19) examined early intervention in patients with severe symptoms post-trauma. One group received CBT, while another received telephone counselling. The telephone counselling group reported significantly fewer post-traumatic symptoms at both 3 months and 1 year, whereas the CBT group showed no significant improvement over time (19). In general, rehabilitation efforts for people with TBI are, in Sweden, rare, and probably less than needed. If problems persist after mTBI contact with a primary healthcare centre is recommended. Access to rehabilitation services for mTBI varies, from specialized to limited care, or no interventions whatsoever (1). A knowledge-based management that describes the Swedish course of care for TBI recommends that patients with high symptom evaluations early post-trauma should be offered individual rehabilitation (20).

A systematic review (21) of 18 RCTs on early intervention for preventing persistent PCS showed mixed results. Seven studies reported positive effects, but due to varied interventions and outcomes, no meta-analysis was conducted. The review concluded that evidence remains inconclusive. Whether early rehabilitation can influence its course and reduce long-term symptoms needs to be evaluated. Before designing a full-scale RCT to investigate the interventions’ effect on health-related outcomes a feasibility testing of the study design, intervention, procedure, and measurements is needed.

The aim was to study feasibility of the study design, intervention, procedure, and measurements in a primary care setting to obtain information regarding whether to proceed with a full-scale RCT.

## METHODS

This study used a randomized controlled pilot study design, was registered on ClinicalTrials.gov 2018 (NCT03771950), and has ethical approval 3 September 2018 from the Ethical Review Committee in Committee in Gothenburg (Dnr 470-18).

Patients diagnosed with mTBI (ICD S06/S06.0) in a county hospital from 31 January 2019 to 31 December 2022 were asked if they would agree to be contacted by telephone 2 to 3 weeks after trauma. If they agreed, they received written information about the study. The inclusion criteria were age 18–65 years, diagnosed with mTBI (ICD S06/S06.0), persistent symptoms or decreased activity, and willingness to participate in the study. The exclusion criteria were no remaining symptoms 2 to 3 weeks post-trauma, return to previous activity and pre-trauma daily living levels, other serious illness, unwillingness to participate in the study, inability to participate in the study protocol, and difficulties with the Swedish language.

Recruitment started on 1 January 2019 with consecutive inclusion until 31 December 2022. The plan was to include 30 participants, but 28 had to be accepted due to slow recruitment.

Approximately 2 weeks post-trauma the first author (Principal Investigator [PI]) contacted participants by telephone. They were asked following question: “Do you have any remaining symptoms related to your head trauma?” and “have you return to previous activity and pre-trauma daily living levels?” If they met the inclusion criteria they were offered participation in this pilot study and time was booked for collecting baseline data.

The PI collected baseline data 2 to 3 weeks after head trauma. This was done either in person or a meeting was arranged either online or by telephone. About 16 weeks after head trauma, post-test data were similarly gathered. After baseline data were collected a sealed envelope based on computer-generated group allocation was opened and group affiliation was announced. The PI was blinded for randomization. It was not possible to blind participants or physio- and occupational therapists for group affiliation.

The intervention group (IG) received rehabilitation by a team consisting of an occupational-, physio-therapist based in primary care and with additional education in neurology and experience of neurological rehabilitation. Participants met the team and described their current problems, anamneses and tests were taken. The rehabilitation team had no access to baseline data. If the physiotherapist could identify oculomotor abnormalities participants received a customized training programme. Rehabilitation was determined through individual goal setting and planning. Participants received advice for physical activity and activities of daily life. The content, frequency, length and intensity of the intervention was depending on needs. The participants’ perceived symptoms guided the degree of recommended activity level and physical exertion. The control group (CG) received care-as-usual (information from the emergency department) and were permitted to seek healthcare and/or rehabilitation on their own.

The *RPQ* (22) is a valid, reliable of self-perceived symptoms after mTBI. RPQ has 16 items, assessed on a 5-point scale: never had symptoms, resolved symptoms, mild, moderate, and severe symptoms (22). RPQ has European reference values for a normal population (23). Higher scores indicate more severe problems.

The *Multidimensional Fatigue Inventory (MFI)* (24) is a valid and reliable measurement consisting of 20 items to measure fatigue. Five dimensions are estimated such as general fatigue, physical fatigue, decreased activity, reduced motivation and mental fatigue. Respondents use a 1 to 5 scale to rate how well statements about fatigue reflect their experiences last week. Scores range from 4 to 20 in each dimension. Higher scores indicate more severe fatigue.

*Activity level is measured with the Swedish version of the Occupational Gaps Questionnaire (OGQ)* (25, 26) in activities of daily living (ADL). OGQ measures participation by comparing what the patient wants to do with what they actually do. OGQ consists of 30 items, with participants answering yes or no to whether they perform the activity and whether they want to (25, 26). In younger middle age (30–49 years) it is normal to have 4 occupational gaps, and in older middle age (50–64 years) it is normal to have 2 (27).

*Leisure Time Physical Activity Instrument (LTPI)* (28) captures the participant’s physical activity level: sedentary, light-, moderate- and high activity level. The subject recalled the average weekly hours spent at the given activity level over the last 4 weeks. The scale was simplified to: 0.5–1.5 h, 2–4 h, and more than 4 h weekly.

*Sleep quantity* was measured by the question: “Do you feel you get enough sleep?”. *Sleep quality* was measured by the question: “Considering everything, how do you feel you sleep?” Participants answered the questions on a 4-grade-scale, higher score indicate better sleep.

*RAND-36* (29) consists of 36 items that measure health-related quality of life comprising eight sub-scales ranging from 0 to 100. The sub-scales are physical function, role physical, bodily pain, general health, vitality, social functioning, role emotional, and mental health. Scale scores are summed and transformed into scales ranging from 0 (worst possible health state) to 100 (best possible state) (29). Reference data from the general population in mid Sweden was used (30).

The *Hospital Anxiety and Depression Scale *(31) was used to register symptoms of anxiety and depression. HADS consists of 14 statements (0–21) for each part, where higher scores indicate more severe anxiety and depression. A score of 0–7 points indicates no bothersome anxiety or depression, 8–11 points suggest mild to moderate anxiety or depression, and 12–21 points indicate a possible anxiety or depression disorder.

### Analysis

All data were analysed descriptively and presented as mean (standard deviation), median (25 and 75 percentile) number and percent depending on data level. Raw differences were calculated between baseline and post-test. Sankey diagrams were used (32) for data visualization for the transition from baseline to post-test for anxiety and depression (HADS). The Statistical Package for Social Science (SPSS) Windows, version 25.0 was used for all analyses.

## RESULTS

**Fig. 1:**
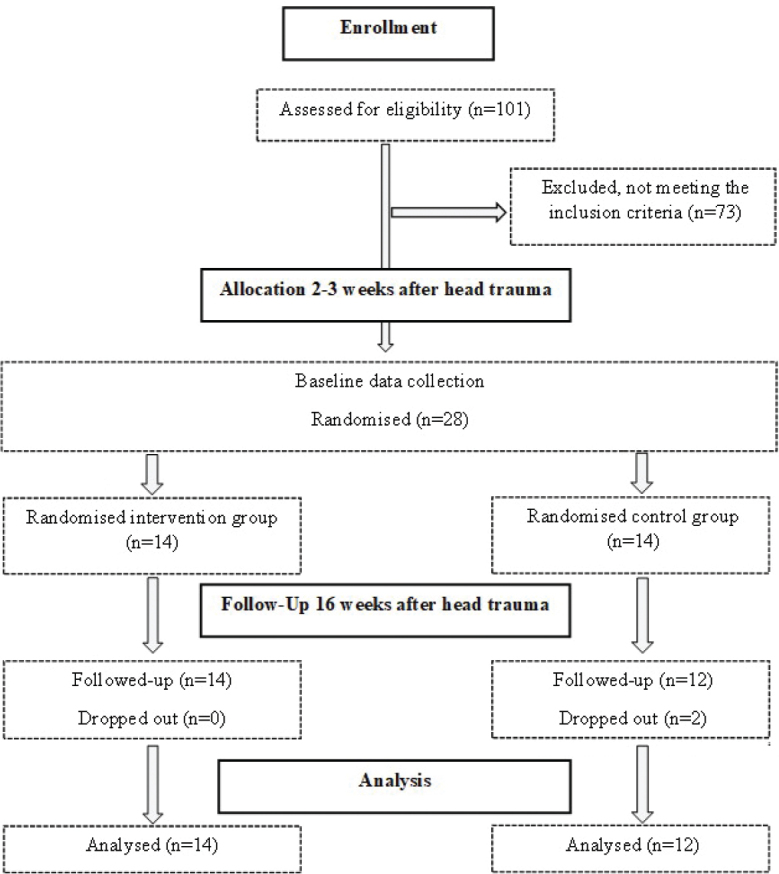
Flowdiagram of the selection process.

A total of 682 patients, 351 women and 331 men were diagnosed with mTBI (ICD S06/S06.0). The PI got contact details to 101 persons and contacted them 2 to 3 weeks after head trauma. Twenty-eight participants were included (Fig.1) and randomized into the IG or CG.

The majority of those included were female (82%). The most common causes of head trauma were falls (57%), 53% of participants had had earlier head trauma, and 39% had different comorbidities in their history. There were no statistically significant differences between groups at baseline (Table I). The IG (*n*=14) were assessed by both a physio- and occupational therapist. During conversations with participants goal setting was carried out along with a plan to achieve these goals. Participants received rehabilitation from 1 or both therapists based on individual needs. Number of visits to the neurological team varied between 1 and 15 visits, depending on the individual’s needs. Two participants continued with the neurological team after the post-test.

**Table I:** Group characteristics at baseline

	Intervention group (IG)	Control group (CG)
Age, Years	45 (12) 47 (34;57)	43 (10) 42 (36;49)
Gender, Female	79% (11/14)	86% (12/14)
Trauma cause		
fall	50% (7/14)	64% (9/14)
sports (ski, snowboard, dive, ride)	7% (1/14)	21% (3/14)
traffic (bicycle, motorcycle, car-accident)	14% (2/14)	14% (2/14)
blow to head	29% (4/14)	-
Previous trauma, Yes	64% (9/14)	43% (6/14)
Comorbidity, Yes	36% (5/14)	43% (6/14)
physical health problem	21% (3/14)	21% (3/14)
psychological health problem	14% (2/14)	14% (2/14)
unclear health problem	-	7% (1/14)
Work ability	64% (9/14)	43% (6/14)
Family status		
living with parents	7% (1/14)	7% (1/14)
living with partner	71% (10/14)	43% (6/14)
living alone	21% (3/14)	50% (7/14)
Employment		
studying	21% (3/14)	21% (3/14)
working	64% (9/14)	79% (11/14)
disability pension	7% (1/14)	0% (0/14)
retired	7% (1/14)	0% (0/14)
Fulltime sick leave at baseline time	64.3% (9/14)	42.9% (6/14)

Data based on interview. Mean value (Standard deviation) Median value (25;75 percentile).

### Health status at baseline and post-test

RPQ mean scores indicated a clinically relevant symptom burden at baseline: IG 26 (SD10), CG 39 (SD12). Both groups showed reductions from baseline to post-test, IG by 4 points, CG by 13 though symptoms burden remained clinically relevant. Fewer symptoms were reported by both groups 16 weeks post-injury compared to baseline (Table II).

**Table II:** Health status in intervention and control groups

Outcome measures	Baseline		Post-test	
Body function	Intervention group (*n*= 14)	Control group (*n *= 14)	Intervention group (*n *= 12)	Control group (*n *= 12)
RPQ total score	26 (10) 27 (23;33)	39 (12) 34 (32;51)	22 (10) 23 (14;33)	26 (18) 25 (9;43)
MFI-20				
general fatigue	14 (3) 14 (12;17)	15 (4) 14 (11;19)	14 (3) 14 (12;17)	15 (5) 14 (11;19)
physical fatigue	14 (5) 15 (11;20)	14 (4) 15 (10;18)	12 (2) 12 (10;14)	11 (3) 12 (9;14)
reduced activity	16 (4) 15 (13;20)	15 (3) 15 (12;18)	12 (5) 13 (6;15)	12 (5) 12 (7;15)
reduced motivation	11 (5) 11 (6;14)	13 (4) 13 (10;15)	8 (3) 8 (6;11)	12 (5) 12 (7;17)
mental fatigue	14 (3) 13 (12;16)	15(4) 16 (14;18)	12 (4) 12 (9;15)	13 (4) 12 (11;17)
Sleep				
quantity	2 (1) 2 (1;3)	2 (1) 2 (2;3)	3 (1) 3 (2;3)	3 (1) 3 (2;3)
quality	2 (1) 2 (2;3)	2 (1) 2 (2;3)	3 (1) 3 (2;3)	3 (1) 3 (2;3)
HADS-anxiety	6 (4) 6 (3;9)	8 (4) 7 (5;12)	6 (4) 5 (3;8)	9 (6) 5 (4;14)
HADS-depression	3 (3) 3 (1;5)	7 (4) 6 (3;10)	4 (3) 4 (1;6)	6 (6) 4 (1;11)
***Activity/participation***				
Physical activity; hours/week				
mostly sedentary	50% (7/14)	57% (8/14)	17% (2/12)	0% (0/12)
light activity; 0,5-1,5	29% (4/14)	36% (5/14)	17% (2/12)	33% (4/12)
light activity; 2-4	29% (4/14)	21% (3/14)	42% (5/12)	17% (2/12)
light activity; >4	21% (3/14)	21% (3/14)	42% (5/12)	50% (6/12)
moderate activity; 0,5-1,5	29% (4/14)	14% (2/14)	33% (4/12)	50% (6/12)
moderate activity; 2-4	14% (2/14)	36% (5/14)	33% (4/12)	17% (2/12)
moderate activity; >4	14% (2/14)	7% (1/14)	25% (3/12)	25% (3/12)
vigorous activity; 0,5-1,5	29% (4/14)	14% (2/14)	50% (6/12)	33% (4/12)
vigorous activity; 2-4	14% (2/14)	21% (3/14)	25% (3/12)	33% (4/12)
vigorous activity; >4	0% (0/14)	7% (1/14)	0% (0/12)	8% (1/12)
***Health-related quality of life***				
RAND-36				
physical function	63 (47-78)	67 (53-81)	75 (63-86)	80 (64-97)
role physical health	14 (-2-31)	17 (-2-36)	32 (4-60)	54 (26-83)
role function emotional	43 (18-67)	44 (15-72)	42 (11-74)	50 (24-76)
energy/fatigue	31 (20-43)	34 (22-46)	42 (32-53)	45 (30-60)
emotional well-being	61 (50-72)	50 (41-60)	68 (54-83)	63 (54-83)
social function	55 (37-68)	48 (27-68)	69 (50-88)	67 (45-89)
pain	28 (14-41)	41 (22-59)	55 (41-68)	66 (51-82)
general health	58 (45-70)	59 (45-74)	56 (44-67)	55 (38-72)
health change last year	32 (17-48)	27 (14-40)	30 (6-53)	40 (22-56)

Mean value (Standard deviation) Median value (25;75 percentile).

MFI: Multidimensional Fatigue Inventory.

MFI-20 scores for both IG and CG showed no change from baseline to post-test for the subscale general fatigue. The sub-scales physical fatigue, decreased activity, reduced motivation and mental fatigue showed decreased fatigue in both groups. At post-test, sleep quantity and quality were improved in both groups (Table II).

**Fig. 2:**
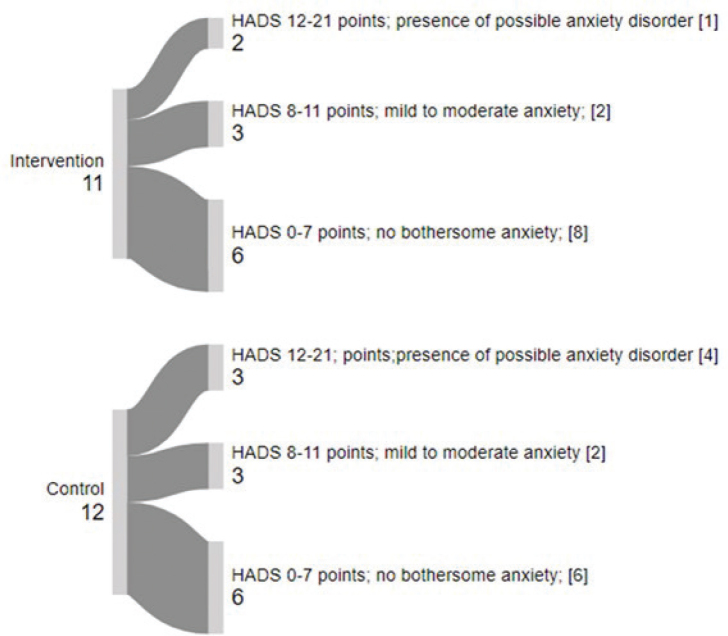
The Hospital Anxiety and Depression Scale (HADS)-anxiety from baseline to post-test. Thickness of arrows represents the number of participants; [Number of participants at baseline]: Number of participants at post-test.

**Fig. 3:**
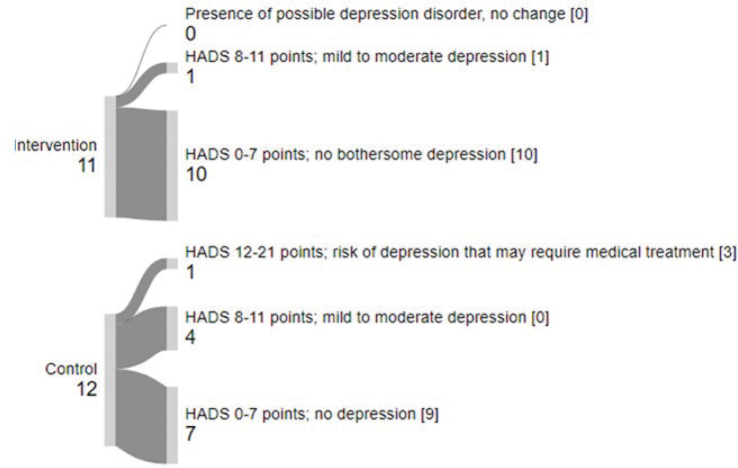
The Hospital Anxiety and Depression Scale (HADS)-depression from baseline to post-test. Arrow thickness represents the number of participants; [Number of participants at baseline]: Number of participants at post-test.

HADS results indicated an increase in depression symptoms in the IG and an increase in anxiety symptoms in the CG from baseline to post-test (Table II). The CG also showed greater variability in depression scores, with both increases and decreases observed across categories (Figs. 2 and 3).

At baseline, a higher proportion of IG participants were classified as “mostly sedentary” compared to the CG. At post-test, sedentary time had decreased in both groups, accompanied by equal increases in light, moderate, and vigorous physical activity (ranging from 0.5 to over 4 h per week). RAND-36 scores improved across all subscales except for general health (Table II).

As the study took place during the COVID-19 pandemic, occupational gaps related to limited social and cultural activities were excluded due to associated restrictions. At baseline, the IG reported an average of 6 gaps, while the CG reported 9. At post-test, the number of gaps had decreased by 53% in the IG (3 gaps) and by 45% in the CG (5 gaps) (Table II).

## DISCUSSION

This pilot study aimed to evaluate the design, procedures, and measurements of a RCT involving participants with mTBI in a primary care setting. Both the IG and CG demonstrated symptom improvement from baseline to post-test across most measures. However, anxiety in the CG and depression symptoms in the IG did not improve. Additionally, general fatigue, as measured by the MFI subscale, remained unchanged. The county hospital emergency department shared contact information for patients with mTBI, although many were missing from the PI. Future RCT need more effective recruitment based on the emergency department.

### Strengths and limitations procedures

The study aimed to recruit 30 participants within 1–2 years but required 4 years to enroll 28 before ending. Of the 682 individuals diagnosed with mTBI at the county hospital, only 101 contact details reached the PI. Around one-third of these had persistent symptoms after 2–3 weeks and were eligible. No participants declined, withdrew, or dropped out.

A total of 581 cases were not communicated. Contributing factors may include limited physician awareness or prioritization of the study, and patient refusal of follow-up 2 weeks post-injury. The COVID-19 pandemic likely further hindered study execution.

Although in-person data collection was planned, COVID-19 restrictions necessitated digital alternatives for some participants. Objectives included evaluating the suitability of the inclusion criteria for individuals with mTBI and the post-test questionnaire completion rate. Seven self-assessment tools were used, typically requiring 30–60 min depending on symptom severity. Some participants found the baseline assessment fatiguing but managed with breaks. All instruments provided valuable insights into self-perceived outcomes following mTBI.

### Strengths and limitations of Patient Reported Outcome Measures (PROMs)

Results from the RPQ in this study showed that participants in both groups reported a decrease in their ratings from baseline to post-test, similar to participants in another Swedish study (33), where the RPQ was also used. That study found that 44% of patients reported 1 or more cognitive symptoms on the first day, 27% after 2 weeks, and 26% at 3 months post-trauma (33). Even though participants in this study showed improvements in their ratings, their scores remained lower than reference values from the general population (mean 14.7, median 12.0) (23).

The RPQ mean scores at baseline indicated a clinically relevant symptom burden in both groups, consistent with the general population reference value of 12 points (SD13). This suggests that participants were experiencing symptoms above normative levels at study entry. Although both the IG and CG showed improvements at post-test, the mean scores remained above the clinical cut-off score, indicating persistent symptom burden (23). These findings highlight the need for continued rehabilitation.

In this study, participants self-rated fatigue using the MFI (24). The “general fatigue” subscale showed no change from baseline to post-test, but mean scores aligned with reference values from healthy medical students and soldiers (24).

Occupational gaps, which reflect discrepancies between desired and performed activities rather than ability, decreased from baseline to post-test in both groups. Reference values for occupational gaps (34) are not directly comparable, as this study included only gaps where participants wished to engage in an activity but could not.

Assessing anxiety and depression is relevant after mTBI, as depression is common in this population (12, 31, 32). Early antidepressant therapy may prevent major depression and is not contraindicated post-injury (35). Proactive detection can enhance mental health, cognition, somatic symptoms, and daily functioning. As shown in Fig. 2, anxiety increased, and fewer participants rated themselves as non-depressed at post-test compared to baseline.

A previous prospective study (12) found that 31% had a psychiatric disorder after 1 year, with 22% being new cases. However, the participants’ pre-mTBI psychological status in this study was unknown.

We have no information about participants’ psychological status before mTBI. A prospective study (12) showed that 31% reported a psychiatric disorder after 1 year and 22% of these participants had developed a psychiatric disorder never previously experienced.

The health measurement methods discussed above have provided valuable information but require further development ahead of a full-scale RCT. The RAND-36 (29) provided valuable data. A reflection is that the items 13–32 assess the past 4 weeks, causing baseline confusion when mTBI occurred 2–3 weeks earlier. However, this problem was not seen at 16 weeks post-test.

Following the pilot study, we believe fewer questionnaires would be preferable, though it’s unclear which to remove. It would have been useful to determine whether occupational gaps were trauma-related or due to other factors. Data on pre-trauma physical activity, sleep issues, and mental health would also have added imported information.

### Strengths and limitation for the intervention

In this study, all participants randomized to the IG received treatment from both an occupational therapist and a physiotherapist. The intervention comprised of individualized, team-based neurorehabilitation, which was implemented successfully within the study framework. The intervention was designed to be flexible and reflect clinical practice, which can be considered as a strength but limits the ability to evaluate the effectiveness of specific components.

A systematic review (36) found cognitive rehab and neurocognitive training to be the most effective interventions for mental health and well-being post-mTBI. In this study, the occupational therapist could provide cognitive rehabilitatio when needed. However, they had no access to psychological or neuropsychological services offering neurocognitive rehabilitation. Limited availability of such professionals in Swedish primary care likely hinders rehabilitation. Future studies could consider the inclusion of various professionals within the rehabilitation team.

In conclusion design, procedures, measurements and interventions were feasible but need refinement for a full-scale study.
